# Genome-wide SNP identification, linkage map construction and QTL mapping for seed mineral concentrations and contents in pea (*Pisum sativum* L.)

**DOI:** 10.1186/s12870-016-0956-4

**Published:** 2017-02-13

**Authors:** Yu Ma, Clarice J Coyne, Michael A Grusak, Michael Mazourek, Peng Cheng, Dorrie Main, Rebecca J McGee

**Affiliations:** 1 Department of Horticulture, Washington State University, Pullman, WA USA; 2 USDA-ARS Plant Germplasm Introduction and Testing, Pullman, WA USA; 3 USDA-ARS Children’s Nutrition Research Center, Houston, TX USA; 4 Department of Plant Breeding and Genetics, Cornell University, Ithaca, NY USA; 5 Department of Plant Sciences, University of Missouri, Columbia, MO USA; 6 USDA-ARS Grain Legume Genetics and Physiology Research, Pullman, WA USA

**Keywords:** Pea, Mineral nutrients, SNP, Linkage map, Comparative analysis, QTL

## Abstract

**Background:**

Marker-assisted breeding is now routinely used in major crops to facilitate more efficient cultivar improvement. This has been significantly enabled by the use of next-generation sequencing technology to identify loci and markers associated with traits of interest. While rich in a range of nutritional components, such as protein, mineral nutrients, carbohydrates and several vitamins, pea (*Pisum sativum* L.), one of the oldest domesticated crops in the world, remains behind many other crops in the availability of genomic and genetic resources. To further improve mineral nutrient levels in pea seeds requires the development of genome-wide tools. The objectives of this research were to develop these tools by: identifying genome-wide single nucleotide polymorphisms (SNPs) using genotyping by sequencing (GBS); constructing a high-density linkage map and comparative maps with other legumes, and identifying quantitative trait loci (QTL) for levels of boron, calcium, iron, potassium, magnesium, manganese, molybdenum, phosphorous, sulfur, and zinc in the seed, as well as for seed weight.

**Results:**

In this study, 1609 high quality SNPs were found to be polymorphic between ‘Kiflica’ and ‘Aragorn’, two parents of an F_6_-derived recombinant inbred line (RIL) population. Mapping 1683 markers including 75 previously published markers and 1608 SNPs developed from the present study generated a linkage map of size 1310.1 cM. Comparative mapping with other legumes demonstrated that the highest level of synteny was observed between pea and the genome of *Medicago truncatula*. QTL analysis of the RIL population across two locations revealed at least one QTL for each of the mineral nutrient traits. In total, 46 seed mineral concentration QTLs, 37 seed mineral content QTLs, and 6 seed weight QTLs were discovered. The QTLs explained from 2.4% to 43.3% of the phenotypic variance.

**Conclusion:**

The genome-wide SNPs and the genetic linkage map developed in this study permitted QTL identification for pea seed mineral nutrients that will serve as important resources to enable marker-assisted selection (MAS) for nutritional quality traits in pea breeding programs.

**Electronic supplementary material:**

The online version of this article (doi:10.1186/s12870-016-0956-4) contains supplementary material, which is available to authorized users.

## Background

Pea (*Pisum sativum* L.), an important pulse crop, is widely grown for human and animal consumption. It is the plant used by Gregor Mendel to illustrate the principle of genetics [1] and has long been considered a good source for protein, carbohydrates, minerals and vitamins. Associated with high nitrogen fixation, pea plays a vital role in the crop rotation system. In recent years, pea yield production worldwide has exceeded ten million tons. In 2014, the major producers of dry peas were Canada (30.4%), China (13.9%), Russia (13.3%), the United States (6.9%) and India (5.3%) (FAOSTAT, 2014).

With an estimated genome size of ~4.3 Gbp [2] and a high repeat component estimated to be between 50% and 60% [3, 4], the improvement of genetic and genomic resources for pea is required for marker-assisted breeding (MAB). MAB, which involves the use of DNA markers to predict trait performance is widely used in crop breeding [5]. Identification of genome-wide markers has undergone a revolutionary transition over the last few years with the advent of low-cost and high-throughput genotyping by sequencing technology [6]. In comparison to traditional marker discovery, GBS can be combined with marker genotyping, allowing marker discovery and genotyping to be completed at the same time. This assay was developed by Elshire et al. [7] and has been used as a tool in linkage mapping, QTL discovery, genomics-assisted breeding, and genomic diversity analysis in a large range of crops, including barley and wheat [8], rice [9], sorghum [10] and switchgrass [11]. While more than fifty-two genetic linkage maps are available for pea [12], eight are high-density SNP-based [13–20] with only one [20] developed using GBS.

Mineral nutrients are inorganic elements essential for plant and animal growth and development [21]. Based on their quantitative requirements, plant mineral nutrients are classified into two groups, macroelements and microelements. Macroelements, generally found in plant tissues in the mg/g dry weight range, include nitrogen, phosphorus, potassium, calcium, magnesium and sulfur. Microelements include boron, copper, iron, chloride, manganese, molybdenum, and zinc, and are found in plants at the μg/g dry weight or lower range. For humans, plant foods are an important source of essential minerals, but unfortunately, mineral deficiencies are a major concern in global health [22] with over two-thirds of the world’s population estimated to experience inadequate intake of one or more mineral nutrients, with more than half considered iron deficient and over 30% zinc deficient [23]. Nutritional deficiencies are especially prevalent in developing countries where people do not have the resources to adequately diversify their diets with vegetables, fruits and animal products. These mineral nutritional deficiencies can lead to stunted growth and development in children, lower resistance to disease, and increased mortality rates [24]. Improving the levels of minerals in foods, through the process of biofortification, has been proposed as a strategy to help combat these dietary deficiencies. Biofortification through traditional plant breeding or biotechnology can be a powerful and sustainable approach to significantly increase nutrient concentrations in crops [25]. Food legumes provide essential nutrients and usually contain higher concentrations of mineral nutrients than do cereals and root crops [26]. Pea is one of the crops targeted for biofortification and has long been recognized as a valuable, nutritious food for the human diet. According to a study conducted with six different cultivars across seven locations by Amarakoon et al. [27], a single serving of cooked pea seeds (100 g fresh weight) can supply 58–68% of the recommended daily allowance (RDA) of iron for male aged from 18 to 50 years, and 26–30% of the RDA of iron for female in the same age group; 36–58% of the RDA of zinc for male, 48–78% of the RDA of zinc for female. The mineral variation within pea germplasm provides the potential to create new pea cultivars with greater mineral density.

To begin to improve levels of mineral nutrients in pea seeds, an understanding of the genetic basis of these traits is required. The accumulation of mineral nutrients in seeds is determined by a series of complex processes that begin with uptake from the rhizosphere, membrane transport in the roots, translocation and redistribution within the plants through the xylem and phloem systems, and import and deposition in the seeds [28]. To date, genes associated with translocation of several elements have been identified in *Arabidopsis thaliana*, but only limited research has been done in pea [23, 29]. Identification of QTLs provides a valuable platform to help identify the genetic basis underlying phenotypic traits. Previous studies on QTLs for mineral nutrients in legumes have been reported on the model legume *Medicago truncatula* [30], common bean [26, 31–35] and *Lotus japonicus* [36]. However, so far, there are only three QTL studies dealing with mineral nutrients in pea, all of which used association studies in diverse populations [37–39]. Kwon et al. [37] discovered ten DNA markers for seven mineral nutrients (Ca, Cu, Fe, K, Mo, Ni and P), while Diapari et al. [38] discovered nine SNP markers associated with iron and two related with zinc in seeds. In addition, Cheng et al. [39] found five SNP markers associated with calcium and magnesium.

Comparative genetics is used to identify syntenic regions controlling traits of interest among closely related species [40]. Within the legumes, the sequenced genomes of *Medicago truncatula*, *Cicer arietinum*, *Phaseolus vulgaris*, and *Lotus japonicus* can be used to transfer knowledge such as trait loci and underlying genes to less studied crops like pea.

The focus of this study was to develop a series of genomic tools to enable mineral improvement in pea through marker-assisted cultivar development. Increasing seed mineral concentration can be influenced by several factors, including seed weight, slow plant growth and low seed yield [36, 41]. Additionally, a previous QTL study of mineral nutrients in *Lotus japonicus* identified several seed mineral concentration QTLs co-localized with QTLs associated with average seed mass. This suggested that higher seed mineral concentrations might be inversely correlated with seed weight [36]. Therefore, to avoid the utilization of loci associated with high seed mineral concentration but low seed weight, this study also assessed QTL for 100-seed weight and seed mineral content. The objectives of this study were to (1) develop genome-wide SNPs using a GBS approach, (2) construct a high-density genetic map using a RIL population, (3) establish comparative maps between pea and the closely related legumes, and (4) identify QTLs associated with seed weight and mineral concentration and content.

## Methods

### Plant materials and DNA extraction

For this study, a cross was made between ‘Aragorn’ (PI 648006) and ‘Kiflica’ (PI 357292). ‘Aragorn’ is an agronomically desirable and widely grown variety with a low to medium concentration of mineral nutrients, while ‘Kiflica’ is a variety with a high concentration of mineral nutrients and less desirable agronomic characteristics [42]. Aragorn seed was provided by Plant Research (NZ) Ltd. Kiflica seed originally collected in Macedonia and donated to the USDA Western Regional Plant Introduction Station by Aladzajkov Lazar in 1970. Kiflica is freely available from the USDA (https://www.ars-grin.gov). The cross was made in Pullman, WA in 2010 and single seed descent was used to get a F_6_ generation consisting of 158 recombinant inbred lines (RILs).

Fresh leaf tissue from each RIL was ground using a Geno/Grinder 2000 (SPEX SamplePrep, Metuchen, NJ) and total DNA were extracted using DNeasy 96 Plant Kit (QIAGEN, Valencia, CA). A NanoDrop ND-1000 spectrophotometer was used to quantify the DNA concentration of each extracted sample following the manufacturer’s instructions (Nano-Drop Technologies, Wilmington, DE).

### SSR and allele specific marker analysis

A total of 114 simple sequence repeat (SSR) primer pairs from the work of Loridon et al. [43] and one *eIF4E* allele specific marker from the work of Smýkal et al. [44] were chosen to anchor this study’s linkage map to the SSR-based map of Loridon et al. [43]. PCR amplifications were performed with 4 ng genomic DNA, 1 × PCR buffer, 1.5 mM MgCl_2_, 0.2 mM dNTPs, 0.05 μM forward primer, 0.25 μM reverse primer, 0.2 μM M13 primers with dyes of FAM, VIC, NED, PET, 0.6 U BIOLASE^TM^ DNA polymerase (Bioline), and 6.76 μl ddH_2_O in a total volume of 12 μl. The cycling conditions included initial denaturization at 95 °C for 5 min, followed by 42 cycles, each of which consisted of 95 °C for 1 min, 56 °C for 1 min, and 72 °C for 1 min. The final extension was at 72 °C for 10 min. These PCR products were analyzed on an ABI 3730 DNA analyzer (Applied Biosystems) and data were scored using GeneMarker software version 2.2.0 (SoftGenetics).

### SNP markers analysis

Two hundred fifty four gene-based SNP markers from the work of Deulvot et al. [13] were selected for use in this study to anchor this linkage map to the previously published gene-based map. The SNP genotyping was analyzed using the Sequenom MassARRAY iPLEX platform [45]. Eight iPLEX assays, each carrying 28–36 SNP markers, were developed with the software SpectroDesinger v3.0 [39].

The iPLEX GOLD reactions consisted of three parts: the iPLEX PCR reaction, the SAP reaction, and the iPLEX Extend reaction. The iPLEX PCR amplifications were performed with 25 ng DNA sample, 1 × PCR buffer, 2 mM MgCl_2_, 0.5 mM dNTPs, 0.1 μM each PCR primer, 1 U Taq DNA polymerase (Bioline), and 1.8 μl ddH_2_O in a total volume of 5 μl. The reaction was performed at 95 °C for 2 min, followed by 45 cycles, each of 95 °C for 30 s, 56 °C for 30 s, and 72 °C for 1 min. The final extension of 72 °C was for 5 min. After the iPLEX PCR, the SAP reaction was performed with 0.17 μl 10 × SAP buffer and 0.5 U SAP enzyme. Then, samples were incubated at 37 °C for 40 min, followed by 85 °C for 5 min. The iPLEX Extend reaction was performed with 1 × iPLEX buffer, iPLEX terminator, a primer mix containing extension primers with a final concentration between 0.625 and 1.5 μM, and 1.35 U iPLEX enzyme. The amplification conditions were performed as follows: 95 °C for 30 s; followed by 40 cycles, each of which consisted of 94 °C for 5 s followed by 5 cycles of 52 °C for 5 s and 80 °C for 5 s; and a final extension at 72 °C for 3 min. Then, 6 mg of resin was added in each well. The iPLEX extension products were dispensed on a SpectroCHIP through a RS1000 Nanodispenser (Sequenom). A matrix-assisted laser desorption/ionization time-of-flight mass spectrometry (MALDI-TOF) mass spectrometer (Sequenom) was then used for the SNP genotyping.

### GBS library construction and SNP identification

The DNA of the 158 lines of the RIL population and the two parents were used to construct GBS libraries [7]. The concentration of genomic DNA was 100 ng/ul. *Ape*KI which recognizes GCWGC (where W = A or T) was used as the restriction enzyme. The libraries were sequenced on an Illumina HiSeq 2000 at the Cornell University Genomics Core Laboratory.

The raw data were analyzed with the universal network enabled analysis kit (UNEAK) pipeline, which was developed for non-reference GBS SNP calling [11]. In this pipeline, the following parameters were used: minimum number of tags was five, error tolerance rate was 0.03, minor allele frequency (MAF) was 0.4, and the sample calling rate was 0.5. The SNPs with unknown or heterozygous genotypes in one or two parents were also removed. Finally, using an in-house perl script, homozygotes for alleles of ‘Agarorn’ were recorded as “A”, homozygotes for alleles of ‘Kiflica’ were recorded as “B”, and heterozygotes were recorded as “H”.

### Linkage map construction

Polymorphic markers from published maps [13, 43] and SNPs from this GBS study with less than 20% missing data per sample were used to construct the linkage map. The genetic linkage map was constructed using OneMap software [46] with LOD values arranged from 3 to 6 and a recombination frequency less than 0.3. The Kosambi mapping function was used to calculate centimorgan distances. The recombination counting and ordering (RECORD) algorithms were used for ordering the markers [47].

### Comparative mapping

Comparative mapping was performed between pea and genetically close legumes. The mapped pea SNP sequences from this study were pairwise aligned using the BLAST algorithm (BLASTN, E value < 1e-10, percentage similarity > = 90%) with the *Medicago truncatula* genome v4.0 (http://www.jcvi.org/medicago/, [48]), the *Cicer arietinum* genome v1.0 (http://cicar.comparative-legumes.org/, [49]), the *Phaseolus vulgaris* genome v1.0 (http://phytozome.jgi.doe.gov, [50]) and the *Lotus japonicus* genome v2.5 (ftp://ftp.kazusa.or.jp/pub/lotus/lotus_r2.5/pseudomolecule/, [51]). The software Circos [52] was used to visualize the synteny between the closely related species. The cM distances on the pea linkage groups were multiplied by 250,000 to match the bp on the chromosomes of the four genomes.

### Phenotyping

The field experiments were established in two locations, Whitlow (46°74’N 117°13’W) and Spillman (46°43’N 117°10’W) farms in Pullman WA in 2014. Plots in each environment were planted in a randomized complete block design with three replications. The seeds from each plot were harvested from pods, cleaned and dried at room temperature. One hundred seeds from each plot were weighed. To analyze mineral concentrations, 20 g of seeds were ground using stainless-steel coffee grinders to homogenize each sample. Then, 0.5 g sub-samples were digested to dryness using concentrated ultra-pure nitric acid and hydrogen peroxide as previously described [53]. Subsequently, the digestates were resuspended in 2% nitric acid. Each sample was analyzed for ten different elements, B, Ca, Fe, K, Mg, Mn, Mo, P, S and Zn, using inductively coupled plasma optical emission spectroscopy (ICP-OES; CIROS ICP Model FCE12; Spectro, Kleve, Germany). The instrument was calibrated with certified standards each day and blanks and certified tissue standards were run to verify the accuracy of the instrument. Mineral content per seed was calculated by multiplying the average sample elemental concentration by average seed weight.

### Statistical and QTL analysis

All the trait data from tissue analysis under the two different environments were analyzed by analysis of variance (ANOVA) using the SAS program PROC MIXED (SAS Institute Inc., Cary, North Carolina, USA). Pearson’s correlations among the quantitative traits were calculated using the SAS program PROC CORR. The broad-sense heritability (*H* ^*2*^) was calculated for each trait as *H* ^*2*^ *=* σ_G_^2^/[σ_G_^2^ + (σ_GE_^2^/*e*) + σ_e_^2^/*re*], where σ_G_^2^ = genotypic variance, σ_GE_^2^ = variance due to interaction between genotype and environment, σ_e_^2^ = error variance, *e* = number of environments, *r* = number of replicates. The variances were calculated by SAS program PROC MIXED with genotypes and environments considered as random effects. The QTL Cartographer 2.5 software [54] was used to identify and locate QTLs using the composite interval mapping (CIM) method by the permutation test (1000 times) at a *P* value of 0.05. The backward regression model was used to get cofactor and walk speed and window size were set to 1 cM and 10 cM respectively. The LOD score threshold for detecting QTLs was set between 3.0 and 3.2 for all the traits. Mapchart (version 2.2) [55] software was used to draw the genetic linkage map and the QTLs.

## Results

### Polymorphism analysis

A total of 40 out of 114 SSR markers from the map of Loridon et al. [43] showed polymorphism between the two parents, ‘Aragorn’ and ‘Kiflica’. Of these, 27 of the SSR markers were successfully amplified and scored in the RIL population. The 254 Sequenom-designable SNP markers from the map of Deulvot et al. [13] were screened among the RILs, along with eight iPLEX assays, of which 50 were polymorphic. SNPs with more than 20% missing data were removed from further study. Finally, 47 informative SNPs were used for linkage map construction. The *eIF4E* allele specific marker was also polymorphic and used in linkage map construction. The sequences of the polymorphic markers are listed in Additional file 1.

### SNP discovery

Two GBS libraries were constructed using 96 barcodes and the *Ape*KI restriction enzyme to generate SNP data on the two parents and the RILs. A total of 384.7 million reads were obtained from the high-throughput sequencing and 349.6 million reads (91% of total raw reads) met the UNEAK pipeline’s quality control. The number of reads per sample ranged from 0.8 million to 5 million reads with an average of two million reads. In the analysis, identical reads were defined as a tag. A total of 45 million tags were identified in the entire set of reads with the number of tags per sample ranging from 130 K to 481 K, for an average of 255 K (Table 1). 1.2 million tags corresponding to 336.2 million reads (87% of total raw reads) met the minimum standard of 5 reads per tag and were used for SNP calling. Following pairwise alignment, 104 K tag pairs were identified. A total of 3,095 SNPs with a MAF > 0.4 and a sample calling rate > 0.5 were called by the UNEAK pipeline. In order to ensure the SNPs were high quality, only SNPs with homozygous genotypes in both parents and with less than 20% missing data per sample were kept for further analysis. High-quality SNPs (1609) were identified and used for linkage map construction. The sequence reads have been uploaded to the NCBI SRA database with accession number SRP092012.

**Table 1:** Minimum, maximum and average good reads and sequence tags per sample analyzed by UNEAK

	Number of good reads	Number of tags
Minimum	799,069	130,208
Maximum	5,006,825	480,936
Average	1,974,933	254,982
Total	349,563,214	45,131,823

### Linkage mapping

**Fig. 1:**
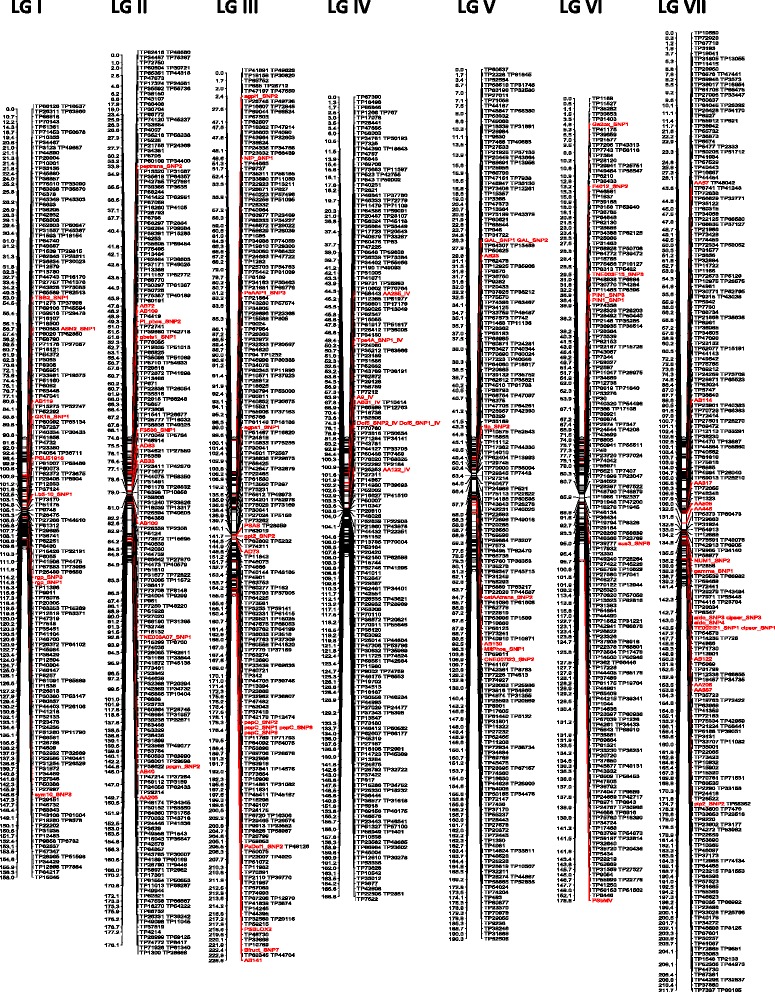
The ‘Aragorn’ × ‘Kiflica’ linkage map. Marker loci are shown on the right and locations are shown on the left. The markers labeled with red color are the anchor markers. The specific details of the linkage map are provided in Additional file 6

The 1609 SNPs identified from GBS were combined with the polymorphic SNP and SSR markers from previous linkage maps to give a set of 1684 markers for constructing the linkage map. Of these markers, only one SNP, TP56850, could not be assigned to a linkage group. 1683 markers were assigned to seven linkage groups with the identity and orientation of the linkage groups determined by the 75 previously mapped markers (Fig. 1). The markers were evenly distributed throughout the seven linkage groups with 99% of the intervals between the adjacent markers being smaller than 10 cM. The estimated map length was 1310.1 cM and the map had a density of 1.3 markers per cM (Table 2).

**Table 2:** Distribution of markers in the ‘Aragorn’ × ‘Kiflica’ genetic linkage map

	LGI	LGII	LGIII	LGIV	LGV	LGVI	LGVII	Total
Number of markers	201	267	261	235	236	229	254	1683
Length (cM)	158	178.1	226.6	168.8	190.3	176.6	211.7	1310.1
Number of marker per cM	1.3	1.5	1.1	1.4	1.2	1.3	1.2	1.3
Number of gaps (>10 cM)	2	3	5	1	2	2	4	19

### Comparative mapping

**Fig. 2:**
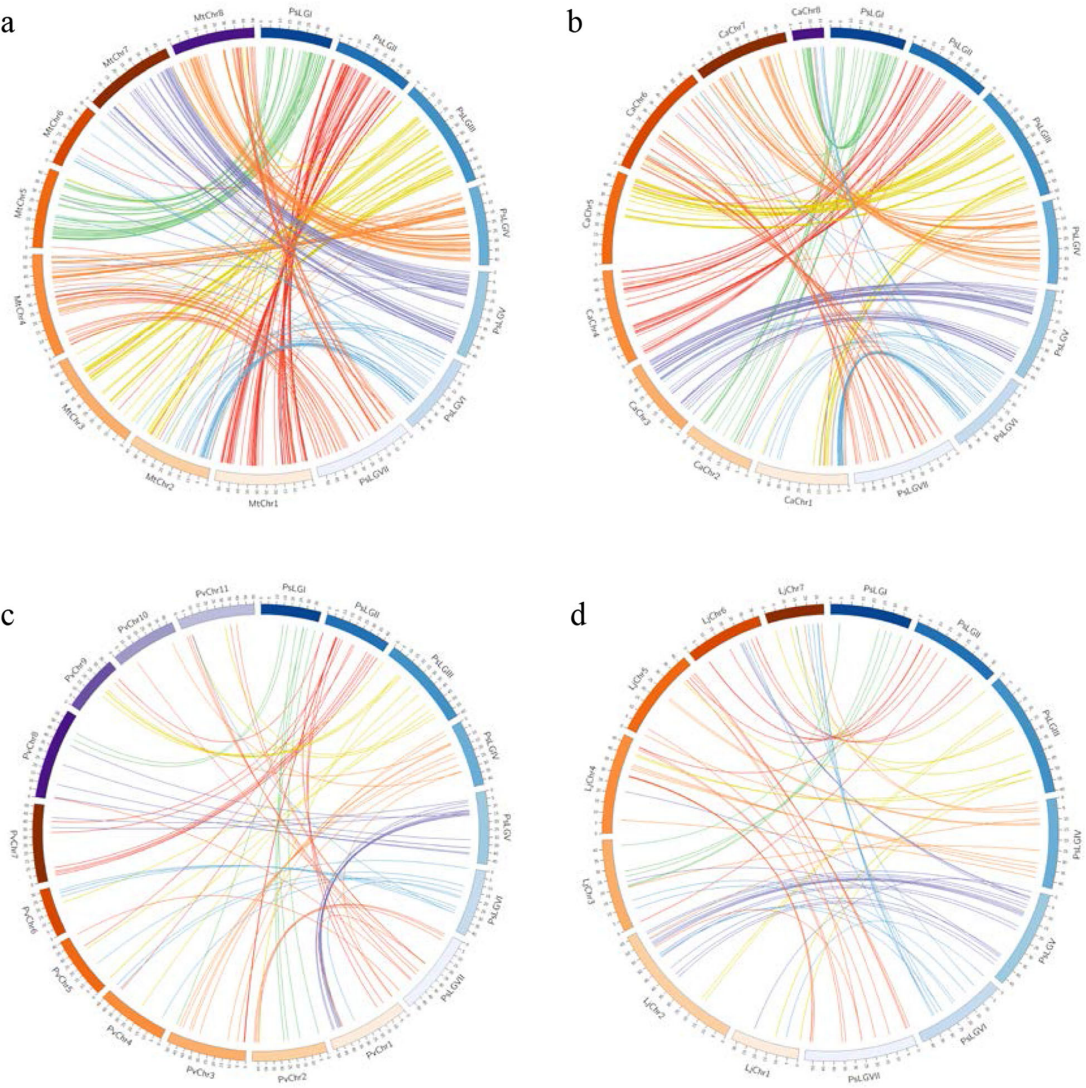
Syntenic relationships of pea linkage groups with other legume chromosomes. **a** Pea LGs shows synteny with the genome assembly of *Medicago truncatula*, (**b**) with *Cicer arietinum* (**c**) with *Phaseolus vulgaris*, and (**d**) with *Lotus japonicus*. The scale unit of the pea linkage groups is cM on the Circos image, while the scale unit of the chromosomes on the other four legumes is Mb

1608 corresponding DNA sequences from the mapped SNP loci were used for comparative genome analysis to evaluate syntenic relationships between pea and other closely related legumes. Comparison between pea and *M. truncatula* showed the closest genetic relationship (402 sequence matches) (Additional file 2). Pea linkage groups PsLG I and PsLG V were syntenic with *M. truncatula* chromosomes MtChr 5 and MtChr 7, respectively. PsLG II exhibited synteny with MtChr 1 with a large inversion. Some pea linkage groups were collinear with more than one *M. truncatula* chromosomes: PsLG III - MtChr 2 and MtChr 3; PsLG IV - MtChr 4 and MtChr 8; PsLG VI - MtChr 2 and MtChr 6; PsLG VII - MtChr 4 and MtChr 8 (Fig. 2a). In the case of pea and chickpea, 296 sequence matches were observed. Among pea linkage groups, PsLG II, PsLG IV, PsLG V and PsLG VII were collinear with *C. arietinum* chromosomes CaChr 4, CaChr 7, CaChr 3 and CaChr 6 respectively. Also, three pea linkage groups, PsLG I, PsLG III and PsLG VI, showed syntenic relationships with CaChr 2 and CaChr 8, CaChr 1 and CaChr 5, CaChr1 and CaChr 8 (Fig. 2b). Although there were 91 sequence matches (Fig. 2c) between pea and *P. vulgaris*, and 86 sequence matches between pea and *L. japonicus* (Fig. 2d), limited syntenic patterns were observed between these genomes.

### Phenotypic analysis

The mean values of mineral nutrient concentration, mineral nutrient content and 100-seed weight for the two parents and the RILs across the two locations are listed in Table 3. Also, the table shows the coefficient of variation and ranges of nutrient concentration, nutrient content and seed weight for the RILs. ‘Kiflica’ had higher nutrient concentration and content than ‘Aragorn’, while ‘Kiflica’ had lower seed weight than ‘Aragorn’. Seed mineral concentration and content ranged from 1.6-fold to 21-fold across the RILs and seed weight varied 2-fold. All the seed traits showed high degrees of correlation between the RILs grown in both locations (Table 3). The P concentration showed the lowest value of correlation (0.27), while Ca concentration showed the highest correlation (0.91). From the frequency distribution histograms shown in Additional file 3, all the traits revealed continuous distribution in two locations and transgressive segregation except for the Fe, Mo and S concentrations. The ANOVA table shown in the Additional file 4 indicates that all the genotypes had significant differences in all the traits. In terms of the environmental effects, there were significant differences (*P* < 0.05) in all the traits with the exceptions of Mn concentration and content, B content and 100-seed weight. Genotype by environment interactions had no significant effect (*P* < 0.05) in most of the mineral concentration traits but showed significant differences (*P* < 0.05) in most of the mineral content traits.

**Table 3:** Statistical analysis of the seed traits for the RILs grown in two locations

		Spillman					Whitlow					
		Aragorn	Kiflica	RILs			Aragorn	Kiflica	RILs			
		Mean	Mean	Mean	CV	Range	Mean	Mean	Mean	CV	Range	Correlation
**Concentration**	**B**	8.4	10.3	9.7	10.6	7.3–12.8	9.0	10.8	9.8	11.3	6.8–13.5	0.60
**(μg/g DW)**	**Ca**	1314.2	1351.0	1313.5	24.1	637.4–2257.2	1217.3	1360.3	1275.4	24.9	521.7–2229.6	0.91
	**Fe**	40.7	53.7	47.2	9.1	37.3–62.0	51.0	66.7	55.3	10.1	40.4–71.2	0.53
	**K**	8471.5	10679.4	9521.5	8.7	7533.2–11884.8	8711.4	11086.6	9803.2	8.5	7583.4–12954.2	0.73
	**Mg**	1284.8	1389.5	1290.9	8.0	955.2–1636.3	1308.3	1574.4	1377.7	8.5	1065.0–1796.7	0.54
	**Mn**	13.2	15.3	15.0	13.3	9.9–21.1	15.1	15.6	14.9	13.9	8.6–20.9	0.72
	**Mo**	0.8	1.3	1.0	44.7	0.3–3.5	0.6	1.3	1.1	55.8	0.2–3.8	0.47
	**P**	3331.5	3578.2	3427.6	12.7	2470.8–5779.1	3826.9	4651.4	4181.5	12.7	2859.4–6013.9	0.27
	**S**	1604.0	2192.4	1963.5	9.8	1513.3–2869.8	1557.6	2177.8	1824.8	10.9	1381.6–2632.0	0.47
	**Zn**	35.7	44.2	41.8	11.3	30.7–61.5	47.1	50.2	47.8	11.0	34.6–64.9	0.32
**Content**	**B**	1.5	1.7	1.7	14.0	1.1–2.5	1.6	1.9	1.8	14.1	1.2–2.7	0.66
**(μg/seed)**	**Ca**	239.0	226.0	235.3	23.7	111.5–384.6	214.7	239.2	227.2	25.1	90.4–407.0	0.86
	**Fe**	7.4	9.0	8.5	14.5	4.7–13.0	9.0	11.7	9.9	15.2	6.3–15.5	0.71
	**K**	1533.6	1788.0	1709.7	10.9	1170.8–2324.5	1538.6	1944.1	1749.1	10.6	1272.5–2295.0	0.66
	**Mg**	232.6	232.8	232.3	12.0	132.3–331.9	230.3	276.2	246.0	11.4	175.1–354.3	0.62
	**Mn**	2.4	2.6	2.7	14.4	1.6–4.0	2.7	2.7	2.7	16.0	1.9–4.3	0.73
	**Mo**	0.1	0.2	0.2	41.4	0.04–0.6	0.1	0.2	0.2	53.2	0.03–0.6	0.43
	**P**	602.7	598.5	616.6	15.8	362.1–904.6	672.2	815.9	747.3	15.4	444.8–1124.9	0.47
	**S**	290.7	367.6	353.5	14.0	204.6–503.0	274.9	382.3	326.8	15.4	203.4–504.5	0.66
	**Zn**	6.5	7.4	7.5	14.9	4.4–11.5	8.3	8.8	8.6	14.9	5.2–13.3	0.58
**100–Seed weight (g)**		18.2	16.8	18.1	12.5	11.9–24.1	17.7	17.5	17.9	12.2	12.7–25.5	0.86

Correlation coefficient analysis was performed between the seed traits among all the RILs in both locations (Additional file 5). Positive correlations were observed between all the seed mineral concentrations and between all the seed mineral contents. Negative correlations were shown between seed weight and all the mineral nutrient concentrations. The highest positive correlations between different mineral concentrations were observed between Ca and Mn (0.69), Mg and Mn (0.69) and the highest positive correlation between different mineral contents was seen between Fe and S (0.85), while the lowest positive correlations between mineral concentrations were between K and Mn (0.13), P and Mo (0.13), Ca and Zn (0.13) and the lowest positive correlations between mineral contents were between Mo and P (0.15), Mo and S (0.15), and Mo and Zn (0.15).

### QTL analysis

QTL analysis was performed for all the seed traits in the ‘Aragorn’ x ‘Kilfica’ RILs across the two locations. A total of 46 QTLs were identified for seed mineral concentrations, 37 QTLs for seed mineral contents, and 6 QTLs for 100-seed weight (Tables 4 and 5). The QTLs were named following the convention of Hamon et al. [56]. The QTLs explained from 2.4 to 43.3% of the phenotypic variance. Co-localizations of QTLs on the seed traits were detected in both locations.

**Table 4:** QTLs for seed mineral concentrations and 100-seed weight in the RILs across the two locations

QTL^a^	Location	LG	Closest marker	Position	LOD	R^2 b^	CI ^c^	Parent allele ^d^
*[B]-Ps1.1*	Whitlow	I	TP26529	138.6	6.7	7.0%	130.6–151.5	Kiflica
	Spillman	I	TP27946	141.6	9.0	10.3%	130.6–151.5	Kiflica
*[B]-Ps5.1*	Whitlow	V	TP61763	40.2	28.3	42.0%	29.6–52.4	Kiflica
	Spillman	V	TP61763	40.2	26.8	41.1%	29.6–52.4	Kiflica
*[B]-Ps6.1*	Whitlow	VI	TP65409	129.2	4.2	4.3%	127.6–131.6	Kiflica
*[B]-Ps7.1*	Whitlow	VII	TP63969	154.8	8.8	9.5%	144.5–164.6	Kiflica
*[B]-Ps7.2*	Spillman	VII	TP6905	134.8	6.7	7.5%	125.5–146.5	Kiflica
*[Ca]-Ps4.1*	Whitlow	IV	TP56003	6.0	9.3	7.4%	0.0–15.8	Aragorn
	Spillman	IV	TP56003	7.0	8.4	7.4%	0.0–15.8	Aragorn
*[Ca]-Ps5.1*	Whitlow	V	TP61763	40.2	28.7	31.0%	29.6–50.1	Kiflica
	Spillman	V	TP61763	40.2	25.0	27.9%	29.6–50.1	Kiflica
*[Ca]-Ps7.1*	Whitlow	VII	AB122_VII	150.3	14.6	12.4%	143.5–164.6	Aragorn
	Spillman	VII	TP8547	142.7	14.5	13.3%	141.4–161.0	Aragorn
*[Ca]-Ps7.2*	Whitlow	VII	TP22498	199.4	3.3	2.4%	199.2–200.2	Aragorn
*[Ca]-Ps7.3*	Spillman	VII	TP44730	208.1	3.4	2.7%	208.1	Aragorn
*[Fe]-Ps2.1*	Whitlow	II	TP50728	44.3	5.0	8.7%	33.9–55.1	Aragorn
	Spillman	II	TP13464	42.1	7.8	15.6%	30.2–51.8	Aragorn
*[Fe]-Ps2.2*	Whitlow	II	TP31957	120.6	3.7	7.5%	118.6–126.6	Aragorn
*[Fe]-Ps5.1*	Whitlow	V	TP61763	40.2	3.9	6.6%	34.1–44.8	Kiflica
	Spillman	V	tip_SNP2_V	43.3	4.8	9.1%	43.3–46.1	Kiflica
*[Fe]-Ps5.2*	Spillman	V	TP40477	54.1	5.0	9.5%	51.4–58.3	Kiflica
*[Fe]-Ps7.1*	Whitlow	VII	TP44143	61.5	9.7	19.4%	52.6–71.5	Kiflica
	Spillman	VII	TP47096	59.9	4.7	9.3%	57.2–67.5	Kiflica
*[K]-Ps3.1*	Spillman	III	TP73262	139.7	3.0	3.8%	139.7	Kiflica
*[K]-Ps4.1*	Whitlow	IV	TP56003	6.0	8.5	10.1%	0.0–15.8	Kiflica
	Spillman	IV	TP56003	6.0	3.1	3.9%	6.0	Kiflica
*[K]-Ps5.1*	Whitlow	V	TP55189	41.5	25.6	43.0%	29.6–52.4	Kiflica
	Spillman	V	TP8375	40.7	20.2	32.7%	31.1–52.4	Kiflica
*[K]-Ps7.1*	Whitlow	VII	TP31577	50.5	5.5	6.3%	44.6–58.9	Kiflica
	Spillman	VII	TP34058	47.5	4.3	5.6%	44.6–59.9	Kiflica
*[K]-Ps7.2*	Whitlow	VII	TP72055	106.3	8.2	9.8%	92.5–120.1	Kiflica
*[K]-Ps7.3*	Spillman	VII	TP35723	154.3	7.9	10.7%	143.5–164.6	Kiflica
*[Mg]-Ps3.1*	Whitlow	III	TP61580	114.0	3.6	5.0%	110.0–120.2	Kiflica
*[Mg]-Ps4.1*	Spillman	IV	TP68325	100.3	3.6	4.8%	98.4–101.6	Kiflica
*[Mg]-Ps5.1*	Whitlow	V	TP61763	40.2	27.0	43.3%	29.6–52.4	Kiflica
	Spillman	V	TP61763	40.2	20.1	35.2%	29.6–50.1	Kiflica
*[Mg]-Ps5.2*	Whitlow	V	TP41157	116.7	3.2	4.7%	115.7–118.8	Kiflica
*[Mn]-Ps1.1*	Whitlow	I	TP13780	32.3	3.2	3.6%	32.3–35.2	Aragorn
	Spillman	I	TP16170	32.7	3.1	3.7%	32.7	Aragorn
*[Mn]-Ps2.1*	Whitlow	II	TP71677	77.1	3.2	3.9%	75.5–78.1	Kiflica
	Spillman	II	F3586_SNP1_II	74.1	8.4	11.4%	61.9–84.8	Kiflica
*[Mn]-Ps4.1*	Whitlow	IV	TP28107	29.0	7.1	12.3%	21.0–43.9	Aragorn
	Spillman	IV	TP28107	31.0	5.9	9.7%	14.9–36.0	Aragorn
*[Mn]-Ps5.1*	Whitlow	V	tip_SNP2_V	44.3	19.4	29.9%	32.5–52.4	Kiflica
	Spillman	V	TP58793	40.5	19.1	29.3%	29.6–50.1	Kiflica
*[Mn]-Ps7.1*	Whitlow	VII	TP35001	161.0	12.5	18.1%	151.9–173.8	Aragorn
	Spillman	VII	TP35001	160.0	3.0	3.7%	160.0	Aragorn
*[Mo]-Ps5.1*	Whitlow	V	TP42330	41.2	16.5	34.2%	31.1–52.4	Kiflica
	Spillman	V	TP42330	41.2	16.5	33.0%	31.1–52.4	Kiflica
*[P]-Ps3.1*	Whitlow	III	TP75231	121.2	7.3	16.9%	108.0–133.2	Aragorn
*[P]-Ps3.2*	Whitlow	III	TP32958	191.3	4.4	8.6%	183.5–194.3	Kiflica
*[P]-Ps5.1*	Whitlow	V	TP61763	40.2	7.5	15.0%	29.6–50.1	Kiflica
	Spillman	V	TP61763	40.2	8.0	14.9%	30.6–50.1	Kiflica
*[P]-Ps7.1*	Whitlow	VII	TP60315	45.0	3.1	5.9%	44.6–45.0	Kiflica
*[P]-Ps7.2*	Spillman	VII	TP40383	90.5	4.4	7.8%	81.6–93.5	Aragorn
*[S]-Ps3.1*	Whitlow	III	TP47722	67.2	8.0	12.6%	57.2–76.2	Kiflica
	Spillman	III	TP74059	65.5	8.6	15.1%	54.5–77.2	Kiflica
*[S]-Ps5.1*	Spillman	V	TP61763	40.2	8.5	14.3%	31.1–52.4	Kiflica
*[S]-Ps5.2*	Whitlow	V	TP27214	53.4	10.0	16.3%	43.3–77.3	Kiflica
*[S]-Ps6.1*	Whitlow	VI	TP41250	147.7	3.7	5.6%	147.7–148.7	Kiflica
	Spillman	VI	TP51502	151.0	4.6	7.6%	140.9–167.1	Kiflica
*[S]-Ps7.1*	Whitlow	VII	TP44143	65.5	4.8	9.4%	51.6–71.5	Kiflica
*[Zn]-Ps2.1*	Whitlow	II	TP31957	125.6	4.5	11.7%	115.9–135.6	Aragorn
*[Zn]-Ps3.1*	Spillman	III	TP2567	102.5	7.6	12.7%	92.4–112.0	Kiflica
*[Zn]-Ps5.1*	Whitlow	V	TP61763	40.2	5.0	9.1%	35.7–42.5	Kiflica
	Spillman	V	TP61763	40.2	6.4	10.4%	29.6–43.3	Kiflica
*[Zn]-Ps7.1*	Whitlow	VII	TP44143	60.5	7.7	14.7%	55.6–71.5	Kiflica
*[Zn]-Ps7.2*	Spillman	VII	TP60315	45.0	3.6	5.6%	44.6–46.0	Kiflica
*SW-Ps3.1*	Whitlow	III	AD73_III	147.8	4.6	9.1%	141.7–165.6	Aragorn
	Spillman	III	AD73_III	147.8	3.1	5.1%	145.9–148.8	Aragorn
*SW-Ps5.1*	Whitlow	V	TP42330	41.2	10.8	20.3%	31.1–52.4	Aragorn
	Spillman	V	TP42330	41.2	13.5	23.2%	31.1–52.4	Aragorn
*SW-Ps5.2*	Spillman	V	TP59611	103.8	3.0	5.0%	103.8	Aragorn
*SW-Ps6.1*	Whitlow	VI	TP45228	95.7	6.2	10.8%	84.7–106.9	Kiflica
*SW-Ps6.2*	Spillman	VI	TP13944	101.9	5.1	7.5%	101.9–120.0	Kiflica
*SW-Ps7.1*	Whitlow	VII	TP65523	81.6	5.2	11.9%	74.6–97.5	Aragorn
	Spillman	VII	TP36649	87.7	6.1	9.4%	76.6–97.5	Aragorn

^a^QTL names represent the traits, the initial of *Pisum sativum*, linkage group # and order of the QTLs

^b^R^2^ is percentage of phenotypic variance explained by the QTL

^c^CI represents 95% confidence interval for the QTL location

^d^Parental allele contributing to the trait

**Table 5:** QTLs for seed mineral contents in the RILs across the two locations

QTL ^a^	Location	LG	Closest marker	Position	LOD	R^2 b^	CI ^c^	Parent allele ^d^
*B-Ps1.1*	Whitlow	I	TP40441	138.3	7.3	15.1%	128.9–148.4	Kiflica
	Spillman	I	TP50388	145.3	6.7	13.7%	130.6–151.5	Kiflica
*B-Ps6.1*	Spillman	VI	TP44490	120.0	4.7	10.3%	113.8–131.6	Kiflica
*B-Ps7.1*	Whitlow	VII	TP65523	81.6	4.5	11.3%	74.6–93.5	Aragorn
	Spillman	VII	TP40383	90.5	5.0	9.7%	78.6–95.5	Aragorn
*Ca-Ps4.1*	Whitlow	IV	TP47685	5.4	5.8	6.5%	0.0–13.9	Aragorn
	Spillman	IV	TP47685	5.4	4.7	5.8%	0.0–10.0	Aragorn
*Ca-Ps5.1*	Whitlow	V	TP27214	53.4	9.9	12.7%	43.3–57.7	Kiflica
	Spillman	V	TP27214	53.4	7.0	9.8%	44.3–56.1	Kiflica
*Ca-Ps7.1*	Whitlow	VII	TP4961	54.6	3.8	4.2%	51.2–54.6	Aragorn
	Spillman	VII	TP4961	54.6	4.8	6.0%	46.0–67.5	Aragorn
*Ca-Ps7.2*	Whitlow	VII	AB122_VII	151.3	17.2	23.1%	141.4–161.0	Aragorn
	Spillman	VII	TP5669	151.9	14.2	20.3%	141.4–161.0	Aragorn
*Fe-Ps2.1*	Spillman	II	TP68770	43.5	5.9	11.9%	33.3–55.1	Aragorn
*Fe-Ps2.2*	Whitlow	II	TP51928	89.9	6.0	12.3%	79.0–98.2	Aragorn
*Fe-Ps3.1*	Whitlow	III	TP75231	135.2	5.2	10.7%	124.2–145.9	Aragorn
*Fe-Ps5.1*	Spillman	V	TP75570	32.5	4.0	7.9%	27.3–41.5	Aragorn
*Fe-Ps6.1*	Whitlow	VI	TP3330	95.4	5.2	10.5%	87.6–99.7	Kiflica
	Spillman	VI	TP36434	91.6	5.3	10.6%	86.6–101.6	Kiflica
*K-Ps3.1*	Whitlow	III	agpl1_SNP2_III	2.4	3.5	7.4%	1.7–8.4	Aragorn
*K-Ps5.1*	Spillman	V	TP8763	126.9	4.2	9.5%	122.9–130.4	Aragorn
*K-Ps6.1*	Whitlow	VI	TP3330	95.4	3.1	6.7%	95.4	Kiflica
*K-Ps6.2*	Spillman	VI	TP25320	113.2	3.3	6.9%	111.2–114.8	Kiflica
*Mg-Ps3.1*	Whitlow	III	TP73169	136.6	3.8	7.7%	132.2–145.9	Aragorn
*Mg-Ps6.1*	Whitlow	VI	TP3330	95.4	7.5	15.9%	82.7–107.9	Kiflica
*Mg-Ps6.2*	Spillman	VI	TP25320	112.2	5.9	13.6%	101.9–123.8	Kiflica
*Mg-Ps7.1*	Whitlow	VII	TP65523	79.6	4.2	11.0%	74.6–94.5	Aragorn
	Spillman	VII	TP66383	93.5	7.4	17.7%	76.6–99.9	Aragorn
*Mn-Ps5.1*	Whitlow	V	TP31866	190.0	3.4	6.2%	188.7–190.0	Kiflica
	Spillman	V	TP39246	188.7	4.2	9.1%	183.4–190.0	Kiflica
*Mn-Ps6.1*	Whitlow	VI	TP45228	95.7	4.6	8.5%	87.6–98.7	Kiflica
*Mn-Ps7.1*	Whitlow	VII	TP73423	162.9	9.6	19.8%	154.3–173.8	Aragorn
	Spillman	VII	TP15320	167.3	4.0	8.6%	162.9–168.3	Aragorn
*Mo-Ps5.1*	Whitlow	V	TP42330	41.2	9.7	21.7%	31.1–52.4	Kiflica
	Spillman	V	TP35621	39.7	11.4	24.6%	29.6–52.4	Kiflica
*P-Ps3.1*	Whitlow	III	TP75231	125.2	6.7	20.5%	110.0–135.2	Aragorn
*P-Ps5.1*	Spillman	V	TP75570	32.5	3.5	6.5%	32.5–35.1	Aragorn
*P-Ps6.1*	Spillman	VI	TP43599	71.5	3.3	6.4%	70.9–72.5	Aragorn
*P-Ps6.2*	Whitlow	VI	TP3330	95.4	3.6	7.7%	95.0–95.7	Kiflica
	Spillman	VI	TP46134	89.6	4.9	10.5%	85.9–97.7	Kiflica
*P-Ps7.1*	Whitlow	VII	TP60315	45.0	3.5	7.5%	43.6–45.0	Kiflica
*P-Ps7.2*	Whitlow	VII	TP66383	92.5	5.6	13.1%	78.6–98.9	Aragorn
	Spillman	VII	TP40383	90.5	10.0	21.0%	77.6–99.9	Aragorn
*S-Ps3.1*	Spillman	III	TP48073	152.7	3.3	6.9%	152.7	Aragorn
*S-Ps6.1*	Whitlow	VI	TP45228	95.7	6.3	14.1%	81.7–99.7	Kiflica
	Spillman	VI	TP45228	95.7	4.4	9.0%	87.6–104.9	Kiflica
*S-Ps7.1*	Spillman	VII	TP40383	90.5	3.5	7.3%	87.7–91.5	Aragorn
*Zn-Ps2.1*	Whitlow	II	TP51928	88.9	3.9	8.1%	84.6–91.5	Aragorn
*Zn-Ps3.1*	Spillman	III	TP30941	102.4	4.2	8.9%	92.4–104.8	Kiflica
*Zn-Ps3.2*	Whitlow	III	TP5158	138.9	6.2	13.7%	121.2–145.9	Aragorn
*Zn-Ps5.1*	Spillman	V	TP60024	38.0	3.4	7.1%	37.5–38.0	Aragorn

^a^QTL names represent the traits, the initial of *Pisum sativum*, linkage group # and order of the QTLs

^b^R^2^ is percentage of phenotypic variance explained by the QTLs

^c^CI represents 95% confidence interval for the QTL location

^d^Parental allele contributing to the trait

Five QTLs were identified for B concentration, two of which were detected in both locations. It is worthwhile to note that the QTL, *[B]-Ps5.1*, explained 42% of the phenotypic variance and had ‘Kiflica’ as the contributing parental allele. Three B content QTLs were observed with explained variances of 15.1%, 10.3%, and 11.3% respectively, two of which had ‘Kiflica’ as the contributing parental allele. Several B concentration QTLs and B content QTLs colocalized with each other: *[B]-Ps1.1* colocalized with *B-Ps1.1*, *[B]-Ps6.1* colocalized with *B-Ps6*.1.

Five Ca concentration QTLs were detected, with the one on LG V, contributed by ‘Kiflica’, explaining 31% of the phenotypic variance. Four Ca content QTLs were identified with all of them observed in both locations. Three Ca concentration QTLs, [*Ca*]*-Ps4.1*, [*Ca*]*-Ps5.1*, [*Ca*]*-Ps7.1*, overlapped with the Ca content QTLs, *Ca-Ps4.1*, *Ca-Ps5.1*, *Ca-Ps7.2*, respectively.

Five Fe concentration QTLs were identified and three of them were observed in both locations. One QTL, [*Fe*]*-Ps7.1*, explained 19.4% of the phenotypic variance with ‘Kiflica’ as the contributing parent. There were five Fe content QTLs detected, four of which had ‘Aragorn’ as the contributing allele. Co-localizations were observed between [*Fe*]*-Ps2.1* and *Fe*-*Ps2.1*, [*Fe*]*-Ps5.1* and *Fe*-*Ps5.1*.

Six K concentration QTLs were identified, three of which were observed in two environments. It is noteworthy that the QTL *[K]-Ps5.1* explained 43% of the phenotypic variance and had ‘Kiflica’ as the contributing allele. Four K content QTLs were observed but none of them was identified in both locations.

Four Mg concentration QTLs and four Mg content QTLs were identified. The concentration QTL *[Mg]-Ps5.1* explained 43.3% of the phenotypic variance and had ‘Kiflica’ as the contributing parent.

Five Mn concentration QTLs were detected in both locations, one of which, *[Mn]-Ps5.1*, explained 29.9% of the phenotypic variance. ‘Kiflica’ was the contributing parent for the QTLs on LG II and LG V, while ‘Aragorn’ was the contributing parent for the QTLs on LG I, LG IV and LG VII. Three Mn content QTLs were identified and one with an explained variance of 19.8% was identified on LG VII. Co-localizations were observed between [*Mn*]*-Ps7.1* and *Mn-Ps7.1*.

The Mo concentration QTL *[Mo]-Ps5.1* on LG V was identified in both locations and it explained 34.2% of the phenotypic variance. The Mo content QTL, *Mo-Ps5.1*, was found with explained the variance of 24.6% and colocalized with the Mo concentration QTL.

Five P concentration QTLs and six P content QTLs were identified. One P content QTL explained 20.5% of the phenotypic variance. Four P concentration QTLs, [*P*]*-Ps3.1*, [*P*]*-Ps5.1*, [*P*]*-Ps7.1*, [*P*]*-Ps7.2*, overlapped with the P content QTLs, *P-Ps3.1*, *P-Ps5.1*, *P-Ps7.1*, P*-Ps7.2*, respectively.

Five QTLs were identified for S concentration, two of which were observed in both locations. All the QTLs for S concentration had ‘Kiflica’ as the contributing parental allele. Three QTLs were detected for S content, one of which explained 14.1% of the variance.

Five Zn concentration QTLs were identified. All the QTLs except the one on LG II had ‘Kiflica’ as the contributing parental allele. Four Zn content QTLs were detected but none of them were identified in both locations. Co-localizations were observed between [*Zn*]*-Ps3.1* and *Zn-Ps3.1*, and [*Zn*]*-Ps5.1* and *Zn-Ps5.1*.

For 100-seed weight, six QTLs were identified, one of which in LG V explained 23.2% of the phenotypic variance and had ‘Aragorn’ as the contributing parental allele. The co-localizations between seed weight and different nutrient traits were observed on LG III, LG V, LG VI and LG VII. The most common co-localization was found on LG V for most of the traits (B, Ca, Fe, K, Mg, Mn, Mo, P, S, Zn concentrations, Ca, Fe, Mo, P, Zn contents and seed weight).

## Discussion

### SNP discovery using GBS

GBS has been shown to be an effective method that allows simultaneous discovery and genotyping of a large number of novel SNPs. The first SNP discovery study using GBS in pea was conducted by Boutet et al. [20] with a total of 64,263 SNPs mapped in a pea genetic map, indicating the GBS approach could significantly facilitate genetic studies and improvement of genomic resources. In our study, a set of 1609 high-quality SNPs were discovered between the two parental lines and well distributed throughout the seven linkage groups of pea. The complexity reduction involving the methylation-sensitive restriction enzyme *Ape*KI was adapted in this study in order to avoid and lower repetitive regions of the genome. The variation of reads per sample observed in some other reported GBS studies [7, 57] was also found in this study. These likely resulted from differences in the DNA quality and quantity among different samples. In this study we provide evidence that *Ape*KI is indeed well-suited for GBS in pea. Alternate restriction enzymes [8] and modified GBS library preparation [58] will provide a further set of tools to customize GBS for SNP discovery in pea. The entire process from DNA extraction, library preparation to next-generation sequencing and SNP calling through the UNEAK pipeline was relatively simple and fast. In addition, the overall cost of GBS was economically efficient. Given the benefits of using GBS as shown here and in other studies [8–11], it may be expected that utilization of this technology will become widely adopted in pea research for marker discovery and application in marker-assisted breeding.

### Genetic linkage mapping

So far, significant efforts have been put into pea genetic linkage map construction and many genetic linkage maps have been successively developed through different types of molecular marker technologies. In this study, the ‘Aragorn’ × ‘Kiflica’ linkage map exhibited a length of 1310.1 cM and an average marker density of 1.3 per cM. It included 1608 SNPs and the 75 anchor markers which were used to determine the orientation of the seven linkage groups. The anchor markers provided a bridge that allowed us to combine our linkage map with the recently published pea consensus map [19]. Comparison of the anchor markers in the ‘Aragorn’ × ‘Kiflica’ map with the previous map [14, 19] revealed high consistency of marker order with only minor exceptions. Such minor inconsistencies might be caused by missing data or chromosome rearrangements [59].

### Comparative mapping

With the availability of sequenced legume genomes, synteny studies can be used to help identify candidate genes underlying specific traits in the less characterized legume species such as pea. Using the mapped pea SNP sequences identified in this study, comparative maps were generated using the whole genome sequences of four legumes from the closely related *M. truncatula* to the distant *L. japonicus.* Common bean and *L. japonicus* belong to the Phaseoleae and Loteae tribes, which are more distantly related to pea, while *M. truncatula* and chickpea belong to the Trifolieae and Cicereae tribes, which are more closely related to pea according to phylogenetic affinities [60]. Consistent with previous comparative mapping studies in pea [15, 19], this study also found pea (~4.3 Gbp [2]) to be genetically closest to *M. truncatula* (~470 Mbp [61]) and chickpea (~740 Mbp [62]). We found that a high level of conservation was observed with comparisons to MtChr 1, MtChr 5, and MtChr 7. In contrast, a low level of conservation was shown in MtChr 2 and MtChr 6, also consistent with previous studies [15, 60]. For common bean and *L. japonicus*, there were fewer large syntenic blocks as compared with *M. truncatula* and chickpea.

### Genetic variation and correlation between traits

Having significant genetic variation in a RIL population is critical in QTL identification studies and cultivar improvement through traditional breeding practices. In this study, while the parents of the RIL population did not exhibit significant differences in some of the mineral nutrients (Ca, Mg, Mn, Mo, P and Zn concentrations; all mineral nutrient contents) large variation was observed for all the mineral nutrients in the RILs, ranging from 1.6-fold to 21-fold. Furthermore, transgressive segregation was observed for most of the traits except Fe, Mo and S concentration and all the traits revealed continuous distributions, which indicated the traits may be controlled by multiple genes, also indicating the potential for mineral nutrient improvement through breeding. Positive and negative correlations are able to be observed in all the traits in a population because of factors such as linkage or environmental differences [63]. It is interesting that positive correlations were observed between all the nutrient concentrations and contents, while the correlations between seed weight and nutrient concentrations were all negative. Similar results were found in the study of *L. japonicus* [36] and common bean [64], where negative correlations were observed between seed weight and Ca, Mg and Mn concentrations. The high positive correlations found between Ca and Mn concentration (0.69), Mg and Mn concentration (0.69), and Fe and S content (0.85), suggests that improving one of these mineral nutrients could simultaneously improve the other.

### Mineral nutrients and seed weight QTLs

In this study, we conducted a comprehensive QTL analysis in a RIL population for pea mineral nutrients (B, Ca, Fe, K, Mg, Mn, Mo, P, S and Zn) to identify genetic loci associated with these traits. A total of 89 QTLs were identified for twenty-one different traits: 46 QTLs for seed mineral concentrations, 37 QTLs for seed mineral contents, and 6 QTLs (*SW-Ps3.1*, *SW-Ps5.1*, *SW-Ps5.2*, *SW-Ps6.1*, *SW-Ps6.2*, *SW-Ps7.1*,) for 100-seed weight. The previous studies on other legumes found 46 QTLs for eight seed mineral concentrations (Ca, Cu, Fe, K, Mg, Mn, P and Zn), 26 QTLs for seven seed mineral contents (Ca, Cu, K, Mg, Mn, P and Zn) and 3 QTLs for seed weight for *M. truncatula* [30]; 34 QTLs for ten seed mineral concentrations (Ca, Cu, Fe, K, Mg, Mn, Ni, P, S and Zn) and 48 QTLs for nine seed mineral contents (Ca, Cu, Fe, K, Mg, Ni, P, S and Zn) for *L. japonicus* [36]; 79 QTLs for ten seed mineral concentrations (B, Ca, Cu, K, Mg, Mn, Na, P, S and Zn) and 9 QTLs for iron and zinc contents for common bean [26, 31–35]. Of the three previously reported association studies for mineral nutrients in pea [37–39] only one [38], which looked at Fe, Zn and Se mineral nutrients, had common markers that could be used to compare co-localization of the corresponding mineral nutrient QTLs identified in this study. On the basis of the common markers between the linkage map from Diapari et al. [38] and the one from the current study, the corresponding positions of markers on these two linkage maps were able to be estimated. Based on common marker Agps1, *[Zn]-Ps3.1* appeared to co-localize with PsC7872p386, a marker associated with Zn concentration on LG III. Three Fe concentration QTLs (*[Fe]-Ps5.1*, *[Fe]-Ps5.2* and *[Fe]-Ps7.1*) appeared to be at the same end of the LG V and LG VII with PsC5316p234 and PsC12961p224. However, the positions of the Fe concentration QTLs in the other two studies [37, 39] could not be precisely estimated because of a lack of common markers. For seed weight, five QTL studies [65–69] have been reported in pea and some QTLs were located in the same linkage groups (LG III, LG V, LG VI, and LG VII) with the QTLs found in the current study. However, it was difficult to estimate if these QTLs co-localized with each other, because of insufficient common markers. Adding common markers between previous and present linkage maps will be needed to facilitate further comparisons of the mineral nutrients and seed weight QTLs among studies.

### QTL co-localization and candidate gene prediction

Co-localizations of the mineral concentration and content QTLs were observed throughout the seven linkage groups in this QTL analysis. Most interesting was the co-localization of B, Ca, Fe, K, Mg, Mn, Mo, P, S, Zn concentration QTLs with Ca, Fe, Mo, P, Zn content QTLs and the seed weight QTL in LG V (29.6–52.4 cM). This finding was not unexpected as previous mineral nutrient QTL studies in other legumes have shown similar co-localizations on *L. japonicus* chromosome 1 [36], *P. vulgaris* chromosomes 1 and 6 [31], and *M. truncatula* chromosomes 1 and 7 [30]. During seed development, seed mineral accumulation relies on continued uptake, translocation, and remobilization of mineral nutrients from different vegetative and reproductive tissues to sink tissues (seeds), with most of these minerals ultimately entering seeds via the phloem pathway [70]. The LG V co-localization region discovered in this study might point to a locus that governs the whole-plant mobilization of several of these minerals (e.g., some global aspect of phloem translocation). Alternatively, for some of the metals, this locus may condition for a metal chelator [71] or a common transporter (e.g., the ZIP transporter protein family members can transport Fe, Mn, and Zn [72] or the CAX transporter family can be bi-functional for Ca and Mn [73]).

To estimate whether co-localizations found in other legumes were syntenic with the co-localization discovered in this study, the comparative maps between those species were developed by mapping the pea SNP sequences to the genome sequences of these legumes. The co-localization of Ca, Cu, Fe, K, Mg, Mn, P, Zn concentration QTLs observed on *M. truncatula* chromosome 7 by Sankaran et al. [30] was found to be syntenic with the LG V mineral co-localization region found in the current study. Putative mineral-related genes located in the vicinity of the co-localization on *M. truncatula* chromosome 7 have been identified, which included Ca^2+^ cation antiporter (*CAX7*), heavy metal transporting P-type ATPases (*HMA*), and the cation/H^+^ exchanger (*CHX*). Also, the co-localization of Fe, Zn concentration QTLs and seed weight QTLs observed on *P. vulgaris* chromosome 1 by Cichy et al. [31] was collinear with the LG V mineral co-localization region in pea. Unfortunately, no putative mineral-related genes were reported in the study of Cichy et al. [31].

In addition, a QTL associated with seed surface (wrinkled/round) with an explained variance of 78.1% was co-localized with the LG V mineral and seed weight co-localization region (unpublished data). This observation was also validated through the comparative mapping between pea and *Medicago truncatula*: a gene coding starch branching enzyme I which controls wrinkled/round seed trait [74] was detected in the LG V mineral co-localization region. Furthermore, a SNP marker Tip_SNP2, located in the LG V mineral co-localization region has significant marker-trait association for the seed surface trait [39]. According to the documented characteristic of pea core collection through the Germplasm Resources Information Network, there is significant difference in terms of seed mineral concentration between round-seed germplasm and wrinkled-seed germplasm: mineral concentrations of wrinkled-seed germplasm are higher than round-seed germplasm. Also, it was reported that there was significant difference in terms of seed weight between round and wrinkled pea [75, 76]: pea seeds with round surface have higher seed weight than pea seeds with wrinkled surface. Although the co-localization was found here in terms of seed surface, seed weight and seed mineral nutrients, further study is needed to confirm a mechanistic association between these traits.

### Choice of QTLs for MAS to increase mineral nutrients

This comprehensive mineral nutrient study provides a foundation of QTLs for use in discovery of markers to improve mineral concentration and content in pea cultivar development. The QTLs, *[B]-Ps5.1*, *[Ca]-Ps5.1*, *[K]-Ps5.1*, *[Mg]-Ps5.1*, and *[Mo]-Ps5.1*, identified in this study explained 42%, 31%, 43%, 43.3%, and 34.2% of phenotypic variations respectively, with ‘Kiflica’ as the parent contributing the high-concentration alleles. Additionally, these QTLs were stable over two environments and were located in a similar region of the genome. This locus is possibly a major-effect region that can be used for improvement of multi-mineral levels together. However, further effort will be necessary before use in breeding. Saturating the linkage map through the addition of more DNA markers will increase the coverage of the whole pea genome and reduce confidence intervals of the QTLs. Furthermore, since QTLs have been reported to be lost or not effective in different population backgrounds [77, 78], it would be important to validate the targeted QTLs in different mapping populations. The future release of a pea reference genome sequence [79] will enable a more complete analysis of these trait loci.

## Conclusion

In this study we developed 1609 high-quality SNPs though GBS, constructed a linkage map and identified 89 QTLs for mineral concentration, content and seed weight in pea. Comparative mapping between pea and four sequenced legumes showed regions of synteny that can be utilized for identifying candidate genes and beneficial alleles to improve mineral nutrient levels in pea cultivar development. The QTLs discovered in this study have potential use in MAS through trait predictions in breeding germplasm. As seed nutrient traits are physiologically complex, further study will be needed in different environments to identify stability of the detected QTLs and identify any additional loci. In conclusion, this study provides a resource for development of tools to enable MAS for mineral nutrients in pea breeding programs, as well as being useful for other researchers working on the genetics of pea.

## Additional files

Additional file 1: Table S1. Information of the polymorphic markers previously published. This file contains the primer information of the polymorphic SNP markers, SSR markers and *eIF4E* allele specific marker. (XLSX 16 kb)
Additional file 2: Table S3. Syntenic relationship. This file shows number of matched loci in the syntenic relationship between pea and *Medicago truncatula*, *Cicer arietinum*, *Phaseolus vulgaris*, *Lotus japonicus (XLSX 12 kb)*

Additional file 3: Figure S1. Frequency distribution histogram. This file contains the frequency distribution histograms of mineral concentration, mineral content and 100-seed weight. The solid bar indicates the Whitlow location and the open bar indicates the Spillman location. “A” indicates the ‘Aragorn’ and “K” indicates the ‘Kiflica’. (DOCX 267 kb)
Additional file 4: Table S4. ANOVA Table This file contains ANOVA table and broad-sense heritability of seed mineral concentration, seed mineral content and 100-seed weight (DOCX 21 kb)
Additional file 5: Table S5. Correlation coefficient analysis Correlation coefficients between different seed mineral concentrations, different seed mineral contents and 100-seed weight. Values below the diagonal are for Spillman and values above the diagonal are for Whitlow. “ns” indicates no significance (DOCX 15 kb)
Additional file 6: Table S2. Linkage map. This file contains the details of the linkage map. (XLSX 38 kb)

## Abbreviations

ANOVA: Analyzed by analysis of variance
*CAX7*: Ca^2+^ cation antiporter
*CHX*: Cation/H^+^ exchanger
CIM: Composite interval mapping
GBS: Genotyping by sequencing
*H*^*2*^: Broad-sense heritability
*HMA*: Heavy metal transporting P-type ATPases
MAF: Minor allele frequency
MALDI-TOF: Matrix-assisted laser desorption/ionization time-of-flight mass spectrometry
MAS: Marker-assisted selection
QTL: Quantitative trait loci
RDA: Recommended daily allowance
RECORD: Recombination counting and ordering
RIL: Recombinant inbred line
SNPs: Single nucleotide polymorphisms
SSR: Simple sequence repeat
UNEAK: Universal network enabled analysis kit
