# Supplementary material for: Genome-wide SNP identification, linkage map construction and QTL mapping for seed mineral concentrations and contents in pea (Pisum sativum L.)
Source: BMC Plant Biol. 2017 Feb 13;17:43. doi: 10.1186/s12870-016-0956-4 (PMC5307697; doi:10.1186/s12870-016-0956-4)
Supplement: Additional file 3: Figure S1. — Frequency distribution histogram. This file contains the frequency distribution histograms of mineral concentration, mineral content and 100-seed weight. The solid bar indicates the Whitlow location and the open bar indicates the Spillman location. “A” indicates the ‘Aragorn’ and “K” indicates the ‘Kiflica’. (DOCX 267 kb) [file 12870_2016_956_MOESM3_ESM.docx]

**Additional file 4: Frequency distributions of mineral nutrient concentration**

K

A/K

K

A

A

A

K

K

A

K

A

K

A

K

K

A

K

A

K

K

A

A

A

K

A

K

A

K

A

K

A

K

A

A

K

K

**Frequency distributions of 100-seed weight**

A/K

K

A

**Frequency distributions of mineral nutrient content**

K

K

K

A/K

A

A/K

K

A

K

K

A

K

K

A

A

A

A

K

K

A

A

A/K

K

A

A/K

K

A

K

K

A

A

A

K

K

A
