# Supplementary material for: Genome-wide SNP identification, linkage map construction and QTL mapping for seed mineral concentrations and contents in pea (Pisum sativum L.)
Source: BMC Plant Biol. 2017 Feb 13;17:43. doi: 10.1186/s12870-016-0956-4 (PMC5307697; doi:10.1186/s12870-016-0956-4)
Supplement: Additional file 4: Table S4. — ANOVA Table This file contains ANOVA table and broad-sense heritability of seed mineral concentration, seed mineral content and 100-seed weight (DOCX 21 kb) [file 12870_2016_956_MOESM4_ESM.docx]

**Additional file 5: ANOVA table and broad-sense heritability of seed nutrient concentration, seed nutrient content and 100-seed weight**

|  |  | **Source** | **DF** | **Sum of square** | **Mean square** | **F Value** | **Pr > F** | ***H^2^*_B_** |
| --- | --- | --- | --- | --- | --- | --- | --- | --- |
| **Concentration**  **(µg/g)** | **B** | Genotype | 157 | 705.26 | 4.49 | 9.11 | <.0001 | 89.4% |
|  |  | Environment | 1 | 3.70 | 3.70 | 7.51 | 0.0068 |  |
|  |  | Genotype*Environment | 157 | 77.46 | 0.49 | 1.15 | 0.1318 |  |
|  |  | Error | 600 | 258.14 | 0.43 |  |  |  |
|  | **Ca** | Genotype | 157 | 84096206.00 | 535645.00 | 54.92 | <.0001 | 98.3% |
|  |  | Environment | 1 | 463979.00 | 463979.00 | 47.60 | <.0001 |  |
|  |  | Genotype*Environment | 157 | 1531160.00 | 9752.61 | 1.04 | 0.3735 |  |
|  |  | Error | 600 | 5635244.00 | 9392.07 |  |  |  |
|  | **Fe** | Genotype | 157 | 13930.00 | 88.73 | 6.04 | <.0001 | 83.9% |
|  |  | Environment | 1 | 14437.00 | 14437.00 | 987.22 | <.0001 |  |
|  |  | Genotype*Environment | 157 | 2306.30 | 14.69 | 1.35 | 0.0068 |  |
|  |  | Error | 600 | 6521.62 | 10.87 |  |  |  |
|  | **K** | Genotype | 157 | 492369589.00 | 3136112.00 | 18.18 | <.0001 | 94.7% |
|  |  | Environment | 1 | 17679740.00 | 17679740.00 | 102.49 | <.0001 |  |
|  |  | Genotype*Environment | 157 | 27078124.00 | 172472.00 | 0.99 | 0.5278 |  |
|  |  | Error | 600 | 104731643.00 | 174553.00 |  |  |  |
|  | **Mg** | Genotype | 157 | 6823859.00 | 43464.00 | 8.37 | <.0001 | 88.6% |
|  |  | Environment | 1 | 1625204.00 | 1625204.00 | 312.32 | <.0001 |  |
|  |  | Genotype*Environment | 157 | 815163.00 | 5192.12 | 0.89 | 0.8214 |  |
|  |  | Error | 600 | 3517429.00 | 5862.38 |  |  |  |
|  | **Mn** | Genotype | 157 | 2912.16 | 18.55 | 14.68 | <.0001 | 93.4% |
|  |  | Environment | 1 | 2.86 | 2.86 | 2.27 | 0.1337 |  |
|  |  | Genotype*Environment | 157 | 198.38 | 1.26 | 1.17 | 0.1028 |  |
|  |  | Error | 600 | 649.19 | 1.08 |  |  |  |
|  | **Mo** | Genotype | 157 | 123.87 | 0.79 | 4.44 | <.0001 | 78.4% |
|  |  | Environment | 1 | 2.13 | 2.13 | 11.91 | 0.0007 |  |
|  |  | Genotype*Environment | 157 | 27.92 | 0.18 | 0.80 | 0.9575 |  |
|  |  | Error | 600 | 133.86 | 0.22 |  |  |  |
|  |  | **Source** | **DF** | **Sum of square** | **Mean square** | **F Value** | **Pr > F** | ***H^2^*_B_** |
|  | **P** | Genotype | 157 | 88495031.00 | 563663.00 | 3.41 | <.0001 | 71.6% |
|  |  | Environment | 1 | 126496130.00 | 126496130.00 | 764.54 | <.0001 |  |
|  |  | Genotype*Environment | 157 | 25963129.00 | 165370.00 | 0.97 | 0.5796 |  |
|  |  | Error | 600 | 102112592.00 | 170188.00 |  |  |  |
|  | **S** | Genotype | 157 | 20454494.00 | 130283.00 | 5.88 | <.0001 | 83.5% |
|  |  | Environment | 1 | 4267352.00 | 4267352.00 | 193.01 | <.0001 |  |
|  |  | Genotype*Environment | 157 | 3481606.00 | 22176.00 | 1.21 | 0.0601 |  |
|  |  | Error | 600 | 10996887.00 | 18328.00 |  |  |  |
|  | **Zn** | Genotype | 157 | 10884.00 | 69.32 | 3.46 | <.0001 | 71.7% |
|  |  | Environment | 1 | 7991.03 | 7991.03 | 400.97 | <.0001 |  |
|  |  | Genotype*Environment | 157 | 3144.28 | 20.03 | 1.40 | 0.0029 |  |
|  |  | Error | 600 | 8584.17 | 14.31 |  |  |  |
| **Content**  **(µg/seed)** | **B** | Genotype | 157 | 40.27 | 0.26 | 10.30 | <.0001 | 87.2% |
|  |  | Environment | 1 | 0.04 | 0.04 | 1.60 | 0.2079 |  |
|  |  | Genotype*Environment | 157 | 3.91 | 0.02 | 1.39 | 0.0033 |  |
|  |  | Error | 599 | 10.72 | 0.02 |  |  |  |
|  | **Ca** | Genotype | 157 | 2563063.00 | 16325.00 | 29.71 | <.0001 | 96.7% |
|  |  | Environment | 1 | 18176.00 | 18176.00 | 33.24 | <.0001 |  |
|  |  | Genotype*Environment | 157 | 86258.00 | 549.42 | 1.38 | 0.0045 |  |
|  |  | Error | 599 | 239212.00 | 399.35 |  |  |  |
|  | **Fe** | Genotype | 157 | 1324.91 | 8.44 | 10.96 | <.0001 | 91.1% |
|  |  | Environment | 1 | 436.35 | 436.35 | 570.73 | <.0001 |  |
|  |  | Genotype*Environment | 157 | 120.85 | 0.77 | 1.64 | <.0001 |  |
|  |  | Error | 599 | 281.99 | 0.47 |  |  |  |
|  | **K** | Genotype | 157 | 23494310.00 | 149645.00 | 10.96 | <.0001 | 91.1% |
|  |  | Environment | 1 | 392302.00 | 392302.00 | 28.90 | <.0001 |  |
|  |  | Genotype*Environment | 157 | 2142966.00 | 13649.00 | 1.45 | 0.0011 |  |
|  |  | Error | 599 | 5626698.00 | 9393.49 |  |  |  |
|  | **Mg** | Genotype | 157 | 500208.00 | 3186.04 | 8.93 | <.0001 | 89.1% |
|  |  | Environment | 1 | 42452.00 | 42452.00 | 119.48 | <.0001 |  |
|  |  | **Source** | **DF** | **Sum of square** | **Mean square** | **F Value** | **Pr > F** | ***H^2^*_B_** |
|  |  | Genotype*Environment | 157 | 56039.00 | 356.94 | 1.36 | 0.0057 |  |
|  |  | Error | 599 | 157015.00 | 262.13 |  |  |  |
|  | **Mn** | Genotype | 157 | 118.10 | 0.75 | 13.60 | <.0001 | 92.9% |
|  |  | Environment | 1 | 0.20 | 0.20 | 3.72 | 0.0556 |  |
|  |  | Genotype*Environment | 157 | 8.68 | 0.06 | 1.39 | 0.0035 |  |
|  |  | Error | 599 | 23.85 | 0.04 |  |  |  |
|  | **Mo** | Genotype | 157 | 3.19 | 0.02 | 4.02 | <.0001 | 76.3% |
|  |  | Environment | 1 | 0.06 | 0.06 | 11.72 | 0.0008 |  |
|  |  | Genotype*Environment | 157 | 0.79 | 0.01 | 0.77 | 0.9782 |  |
|  |  | Error | 599 | 3.95 | 0.01 |  |  |  |
|  | **P** | Genotype | 157 | 5803054.00 | 36962.00 | 6.01 | <.0001 | 84.0% |
|  |  | Environment | 1 | 3839710.00 | 3839710.00 | 625.06 | <.0001 |  |
|  |  | Genotype*Environment | 157 | 964851.00 | 6145.55 | 1.03 | 0.4126 |  |
|  |  | Error | 599 | 3590942.00 | 5994.89 |  |  |  |
|  | **S** | Genotype | 157 | 1680420.00 | 10703.00 | 10.22 | <.0001 | 90.5% |
|  |  | Environment | 1 | 151490.00 | 151490.00 | 145.47 | <.0001 |  |
|  |  | Genotype*Environment | 157 | 164396.00 | 1047.11 | 1.47 | 0.0008 |  |
|  |  | Error | 599 | 427952.00 | 714.44 |  |  |  |
|  | **Zn** | Genotype | 157 | 889.20 | 5.66 | 7.60 | <.0001 | 87.2% |
|  |  | Environment | 1 | 240.99 | 240.99 | 324.98 | <.0001 |  |
|  |  | Genotype*Environment | 157 | 117.05 | 0.75 | 1.44 | 0.0014 |  |
|  |  | Error | 599 | 310.18 | 0.52 |  |  |  |
| **100-seed weight** | **100-seed weight** | Genotype | 157 | 4011.65 | 25.55 | 25.36 | <.0001 | 96.0% |
|  |  | Environment | 1 | 1.96 | 1.96 | 1.96 | 0.1632 |  |
|  |  | Genotype*Environment | 157 | 158.18 | 1.01 | 1.92 | <.0001 |  |
|  |  | Error | 599 | 314.78 | 0.53 |  |  |  |
