# Supplementary material for: Genome-wide SNP identification, linkage map construction and QTL mapping for seed mineral concentrations and contents in pea (Pisum sativum L.)
Source: BMC Plant Biol. 2017 Feb 13;17:43. doi: 10.1186/s12870-016-0956-4 (PMC5307697; doi:10.1186/s12870-016-0956-4)
Supplement: Additional file 5: Table S5. — Correlation coefficient analysis Correlation coefficients between different seed mineral concentrations, different seed mineral contents and 100-seed weight. Values below the diagonal are for Spillman and values above the diagonal are for Whitlow. “ns” indicates no significance (DOCX 15 kb) [file 12870_2016_956_MOESM5_ESM.docx]

**Additional file 6: Correlation coefficient analysis**

**Correlation coefficients between seed nutrient concentrations and 100-seed weight**

|  | [B] | [Ca] | [Fe] | [K] | [Mg] | [Mn] | [Mo] | [P] | [S] | [Zn] | Seed weight |
| --- | --- | --- | --- | --- | --- | --- | --- | --- | --- | --- | --- |
| [B] | 1.00 | 0.34 | 0.24 | 0.41 | 0.61 | 0.37 | 0.39 | 0.27 | 0.37 | 0.17 | -0.29 |
| [Ca] | 0.38 | 1.00 | 0.19 | -0.03 ^ns^ | 0.64 | 0.64 | 0.24 | 0.16 | 0.23 | 0.13 | -0.22 |
| [Fe] | 0.37 | 0.22 | 1.00 | 0.17 | 0.42 | 0.36 | 0.23 | 0.42 | 0.60 | 0.64 | -0.09 ^ns^ |
| [K] | 0.44 | -0.06 ^ns^ | 0.26 | 1.00 | 0.34 | 0.13 | 0.28 | 0.36 | 0.17 | 0.21 | -0.51 |
| [Mg] | 0.55 | 0.64 | 0.48 | 0.31 | 1.00 | 0.69 | 0.30 | 0.40 | 0.38 | 0.36 | -0.43 |
| [Mn] | 0.43 | 0.69 | 0.28 | 0.21 | 0.65 | 1.00 | 0.17 | 0.16 | 0.22 | 0.24 | -0.27 |
| [Mo] | 0.47 | 0.32 | 0.30 | 0.41 | 0.32 | 0.29 | 1.00 | 0.13 | 0.09 ^ns^ | 0.16 | -0.26 |
| [P] | 0.39 | 0.33 | 0.47 | 0.34 | 0.49 | 0.28 | 0.32 | 1.00 | 0.32 | 0.62 | -0.23 |
| [S] | 0.50 | 0.25 | 0.68 | 0.34 | 0.46 | 0.27 | 0.39 | 0.53 | 1.00 | 0.49 | -0.12 |
| [Zn] | 0.38 | 0.14 | 0.61 | 0.37 | 0.37 | 0.31 | 0.34 | 0.48 | 0.56 | 1.00 | -0.16 |
| Seed weight | -0.28 | -0.27 | -0.10 | -0.51 | -0.36 | -0.38 | -0.34 | -0.17 | -0.20 | -0.20 | 1.00 |

Values below the diagonal are for Spillman and values above the diagonal are for Whitlow

**Correlation coefficients between seed nutrient contents**

|  | B | Ca | Fe | K | Mg | Mn | Mo | P | S | Zn |
| --- | --- | --- | --- | --- | --- | --- | --- | --- | --- | --- |
| B | 1.00 | 0.40 | 0.57 | 0.62 | 0.76 | 0.54 | 0.24 | 0.49 | 0.64 | 0.50 |
| Ca | 0.41 | 1.00 | 0.31 | 0.08 ^ns^ | 0.60 | 0.65 | 0.17 | 0.25 | 0.35 | 0.25 |
| Fe | 0.68 | 0.31 | 1.00 | 0.61 | 0.74 | 0.60 | 0.11 ^ns^ | 0.66 | 0.80 | 0.82 |
| K | 0.67 | 0.02 ^ns^ | 0.67 | 1.00 | 0.60 | 0.37 | 0.09 ^ns^ | 0.60 | 0.58 | 0.58 |
| Mg | 0.76 | 0.56 | 0.80 | 0.64 | 1.00 | 0.77 | 0.11 ^ns^ | 0.62 | 0.70 | 0.66 |
| Mn | 0.59 | 0.67 | 0.56 | 0.40 | 0.72 | 1.00 | 0.06 ^ns^ | 0.38 | 0.50 | 0.49 |
| Mo | 0.25 | 0.22 | 0.09 ^ns^ | 0.16 | 0.08 ^ns^ | 0.13 | 1.00 | 0.04 ^ns^ | 0.02 ^ns^ | 0.07 ^ns^ |
| P | 0.63 | 0.38 | 0.71 | 0.63 | 0.74 | 0.49 | 0.15 | 1.00 | 0.58 | 0.76 |
| S | 0.73 | 0.32 | 0.85 | 0.67 | 0.76 | 0.52 | 0.15 | 0.72 | 1.00 | 0.72 |
| Zn | 0.63 | 0.23 | 0.80 | 0.65 | 0.69 | 0.53 | 0.15 | 0.68 | 0.76 | 1.00 |

Values below the diagonal are for Spillman and values above the diagonal are for Whitlow
